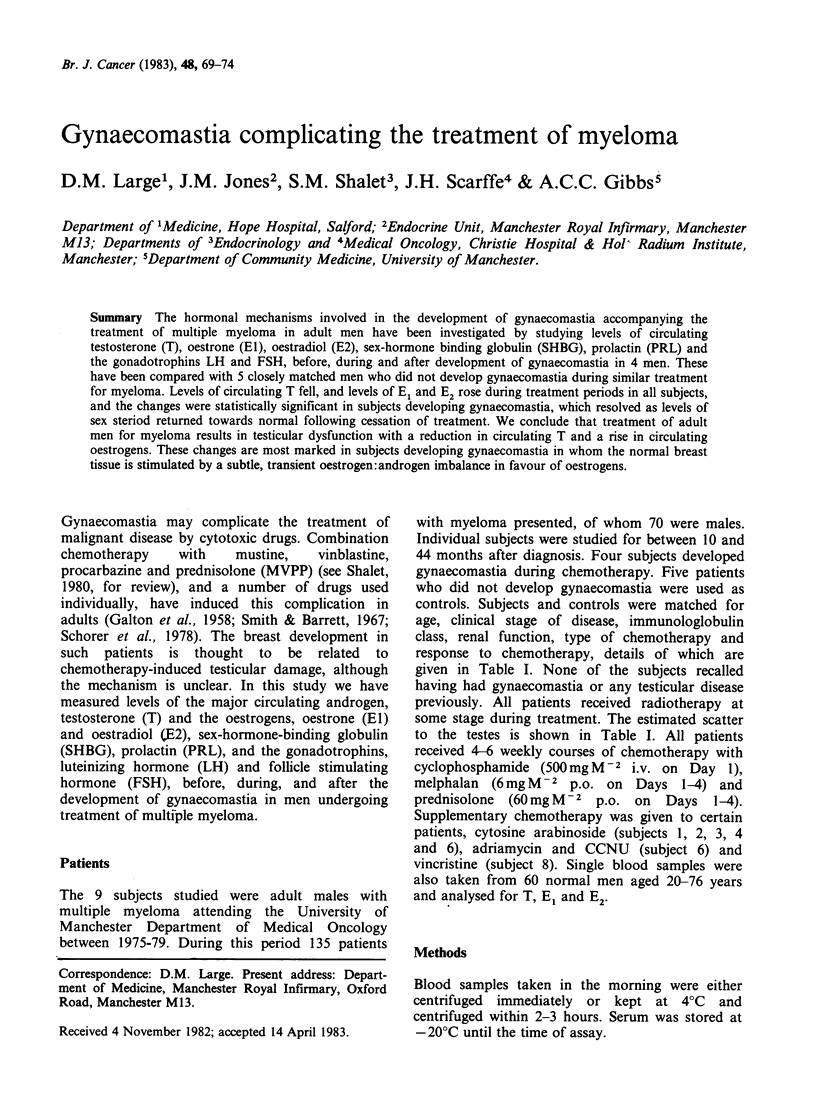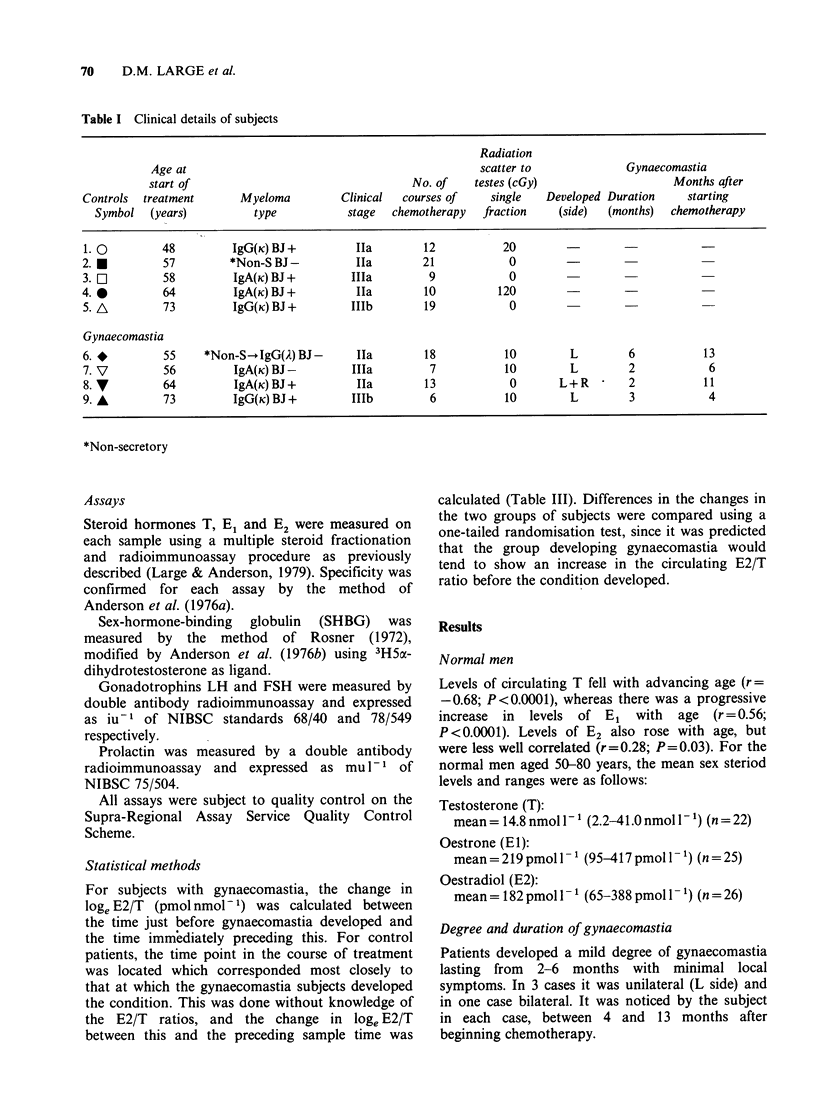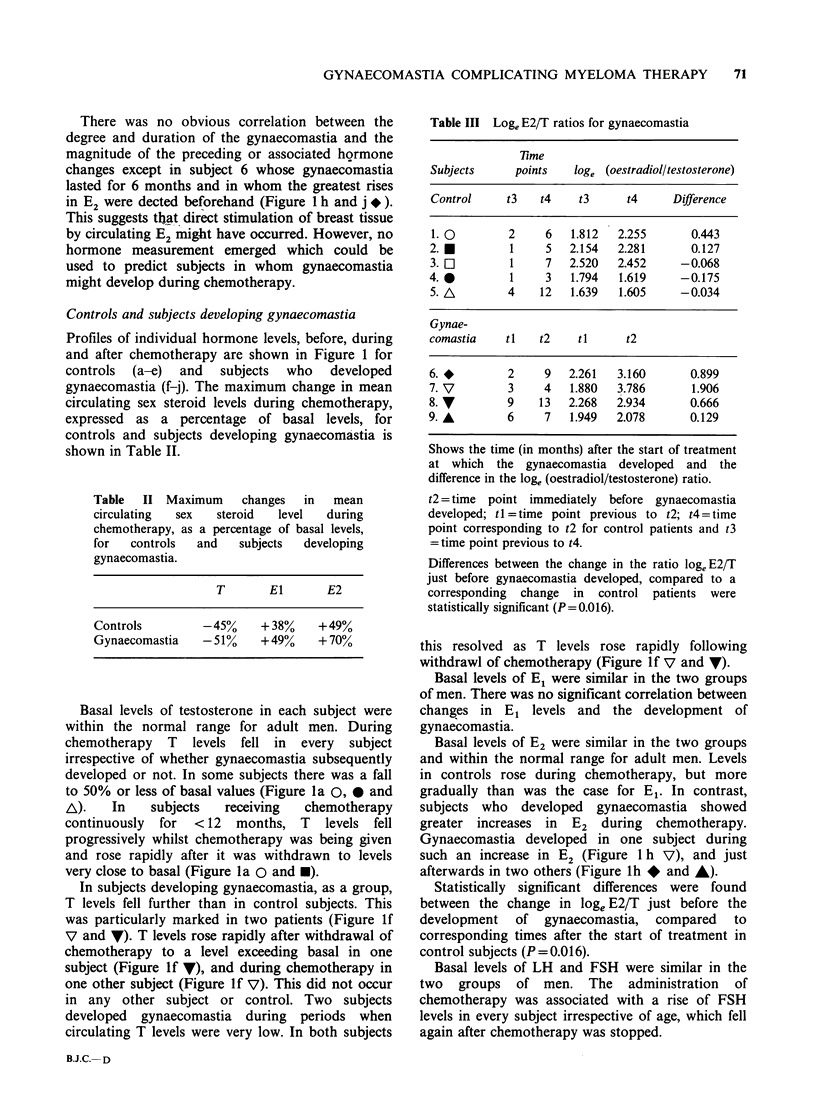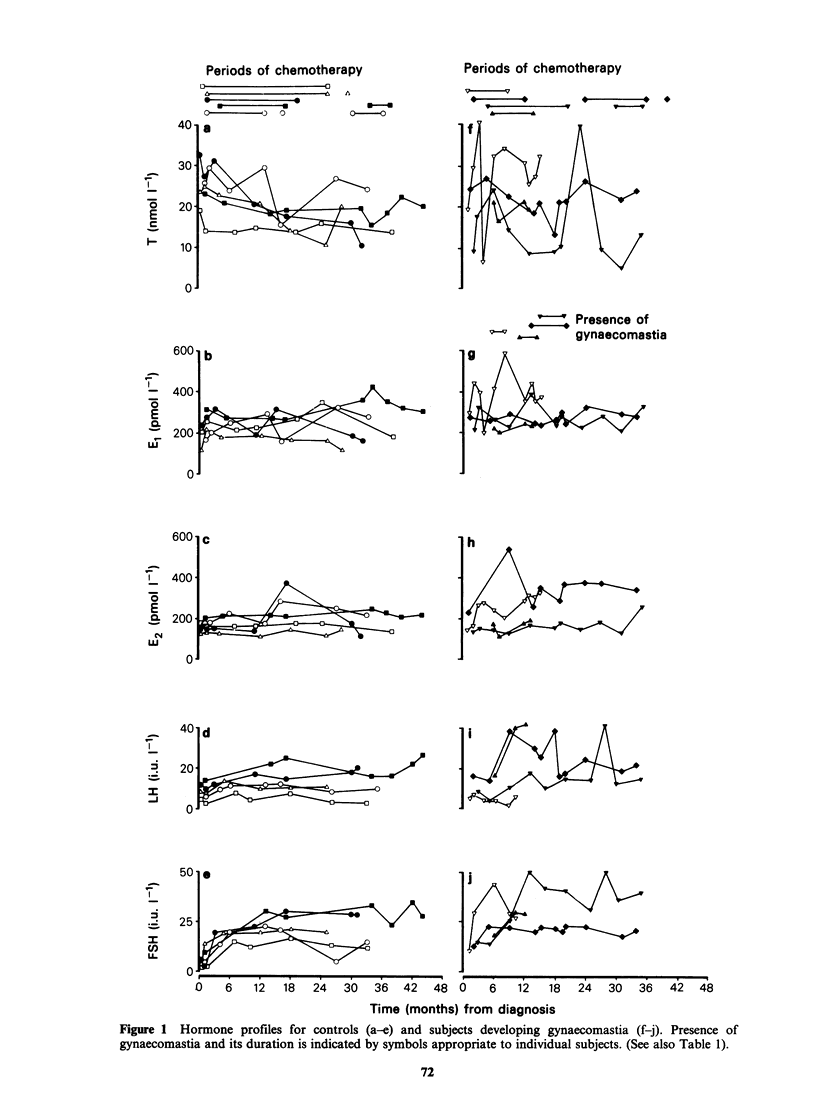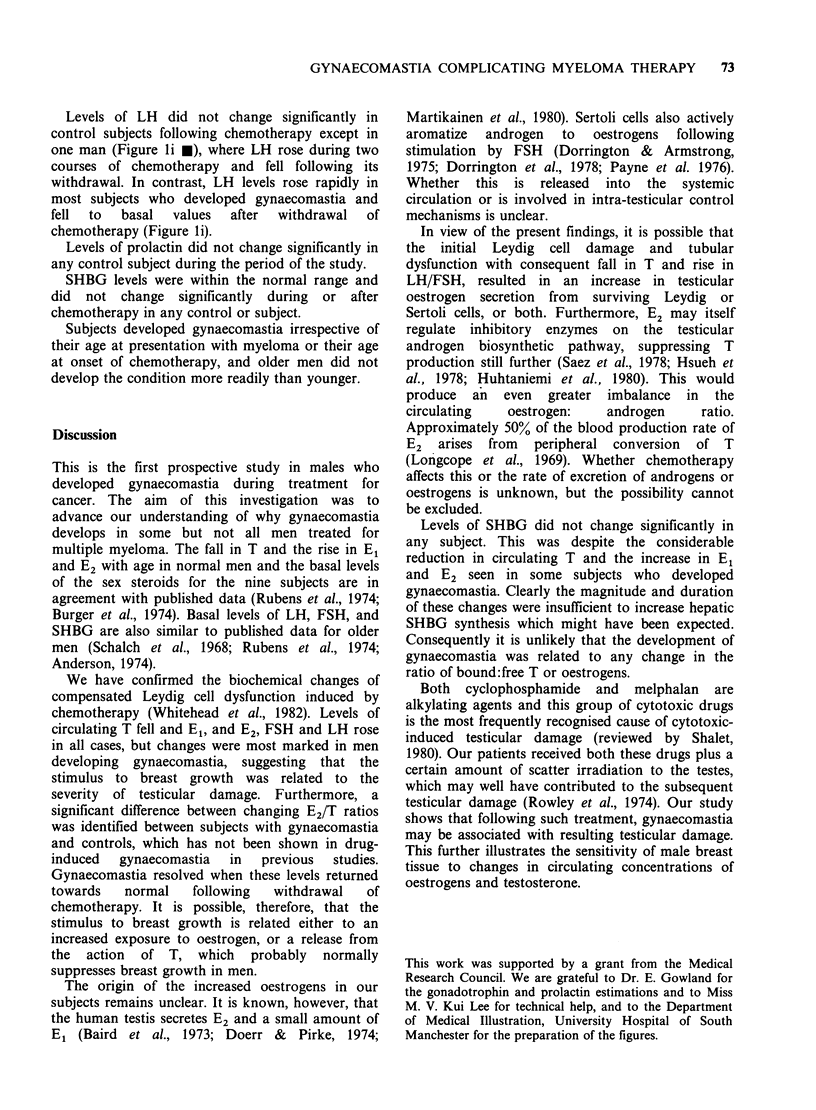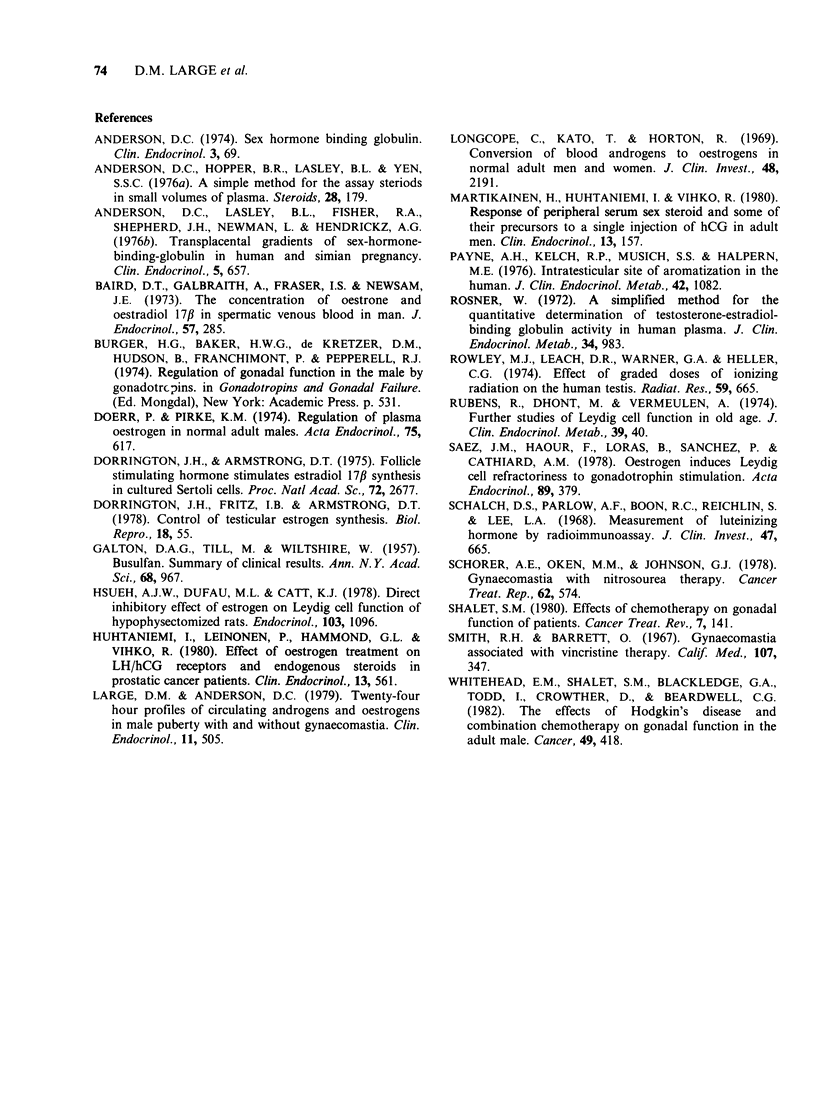# Gynaecomastia complicating the treatment of myeloma.

**DOI:** 10.1038/bjc.1983.158

**Published:** 1983-07

**Authors:** D. M. Large, J. M. Jones, S. M. Shalet, J. H. Scarffe, A. C. Gibbs

## Abstract

The hormonal mechanisms involved in the development of gynaecomastia accompanying the treatment of multiple myeloma in adult men have been investigated by studying levels of circulating testosterone (T), oestrone (EI), oestradiol (E2), sex-hormone binding globulin (SHBG), prolactin (PRL) and the gonadotrophins LH and FSH, before, during and after development of gynaecomastia in 4 men. These have been compared with 5 closely matched men who did not develop gynaecomastia during similar treatment for myeloma. Levels of circulating T fell, and levels of E1 and E2 rose during treatment periods in all subjects, and the changes were statistically significant in subjects developing gynaecomastia, which resolved as levels of sex steroid returned towards normal following cessation of treatment. We conclude that treatment of adult men for myeloma results in testicular dysfunction with a reduction in circulating T and a rise in circulating oestrogens. These changes are most marked in subjects developing gynaecomastia in whom the normal breast tissue is stimulated by a subtle, transient oestrogen:androgen imbalance in favour of oestrogens.


*Br. J. Cancer* (1983), **48**, 69–74

### Gynaecomastia complicating the treatment of myeloma

D.M. Large[1], J.M. Jones[2], S.M. Shalet[3], J.H. Scarffe[4] & A.C.C. Gibbs[5]

*Department of [1]Medicine, Hope Hospital, Salford; [2]Endocrine Unit, Manchester Royal Infirmary, Manchester M13; Departments of [3]Endocrinology and [4]Medical Oncology, Christie Hospital & Hol' Radium Institute, Manchester; [5]Department of Community Medicine, University of Manchester.*

**Summary** The hormonal mechanisms involved in the development of gynaecomastia accompanying the treatment of multiple myeloma in adult men have been investigated by studying levels of circulating testosterone (T), oestrone (E1), oestradiol (E2), sex-hormone binding globulin (SHBG), prolactin (PRL) and the gonadotrophins LH and FSH, before, during and after development of gynaecomastia in 4 men. These have been compared with 5 closely matched men who did not develop gynaecomastia during similar treatment for myeloma. Levels of circulating T fell, and levels of $E_1$ and $E_2$ rose during treatment periods in all subjects, and the changes were statistically significant in subjects developing gynaecomastia, which resolved as levels of sex steriod returned towards normal following cessation of treatment. We conclude that treatment of adult men for myeloma results in testicular dysfunction with a reduction in circulating T and a rise in circulating oestrogens. These changes are most marked in subjects developing gynaecomastia in whom the normal breast tissue is stimulated by a subtle, transient oestrogen:androgen imbalance in favour of oestrogens.

Gynaecomastia may complicate the treatment of malignant disease by cytotoxic drugs. Combination chemotherapy with mustine, vinblastine, procarbazine and prednisolone (MVPP) (see Shalet, 1980, for review), and a number of drugs used individually, have induced this complication in adults (Galton *et al.*, 1958; Smith & Barrett, 1967; Schorer *et al.*, 1978). The breast development in such patients is thought to be related to chemotherapy-induced testicular damage, although the mechanism is unclear. In this study we have measured levels of the major circulating androgen, testosterone (T) and the oestrogens, oestrone (E1) and oestradiol (E2), sex-hormone-binding globulin (SHBG), prolactin (PRL), and the gonadotrophins, luteinizing hormone (LH) and follicle stimulating hormone (FSH), before, during, and after the development of gynaecomastia in men undergoing treatment of multiple myeloma.

### Patients

The 9 subjects studied were adult males with multiple myeloma attending the University of Manchester Department of Medical Oncology between 1975-79. During this period 135 patients with myeloma presented, of whom 70 were males. Individual subjects were studied for between 10 and 44 months after diagnosis. Four subjects developed gynaecomastia during chemotherapy. Five patients who did not develop gynaecomastia were used as controls. Subjects and controls were matched for age, clinical stage of disease, immunologlobulin class, renal function, type of chemotherapy and response to chemotherapy, details of which are given in Table I. None of the subjects recalled having had gynaecomastia or any testicular disease previously. All patients received radiotherapy at some stage during treatment. The estimated scatter to the testes is shown in Table I. All patients received 4–6 weekly courses of chemotherapy with cyclophosphamide (500 mg $M^{-2}$ i.v. on Day 1), melphalan (6 mg $M^{-2}$ p.o. on Days 1–4) and prednisolone (60 mg $M^{-2}$ p.o. on Days 1–4). Supplementary chemotherapy was given to certain patients, cytosine arabinoside (subjects 1, 2, 3, 4 and 6), adriamycin and CCNU (subject 6) and vincristine (subject 8). Single blood samples were also taken from 60 normal men aged 20–76 years and analysed for T, $E_1$ and $E_2$.

### Methods

Blood samples taken in the morning were either centrifuged immediately or kept at 4°C and centrifuged within 2–3 hours. Serum was stored at −20°C until the time of assay.

Correspondence: D.M. Large. Present address: Department of Medicine, Manchester Royal Infirmary, Oxford Road, Manchester M13.

Received 4 November 1982; accepted 14 April 1983.

**Table I** Clinical details of subjects

| Controls Symbol | Age at start of treatment (years) | Myeloma type | Clinical stage | No. of courses of chemotherapy | Radiation scatter to testes (cGy) single fraction | Gynaecomastia Developed (side) | Duration (months) | Months after starting chemotherapy |
|---|---|---|---|---|---|---|---|---|
| 1. ○ | 48 | IgG(κ) BJ+ | IIa | 12 | 20 | — | — | — |
| 2. ■ | 57 | *Non-S BJ− | IIa | 21 | 0 | — | — | — |
| 3. □ | 58 | IgA(κ) BJ+ | IIIa | 9 | 0 | — | — | — |
| 4. ● | 64 | IgA(κ) BJ+ | IIa | 10 | 120 | — | — | — |
| 5. △ | 73 | IgG(κ) BJ+ | IIIb | 19 | 0 | — | — | — |
| *Gynaecomastia* | | | | | | | | |
| 6. ◆ | 55 | *Non-S→IgG(λ) BJ− | IIa | 18 | 10 | L | 6 | 13 |
| 7. ▽ | 56 | IgA(κ) BJ− | IIIa | 7 | 10 | L | 2 | 6 |
| 8. ▼ | 64 | IgA(κ) BJ+ | IIa | 13 | 0 | L+R | 2 | 11 |
| 9. ▲ | 73 | IgG(κ) BJ+ | IIIb | 6 | 10 | L | 3 | 4 |

*Non-secretory

### *Assays*

Steroid hormones T, $E_1$ and $E_2$ were measured on each sample using a multiple steroid fractionation and radioimmunoassay procedure as previously described (Large & Anderson, 1979). Specificity was confirmed for each assay by the method of Anderson *et al.* (1976*a*).

Sex-hormone-binding globulin (SHBG) was measured by the method of Rosner (1972), modified by Anderson *et al.* (1976*b*) using $^3$H5α-dihydrotestosterone as ligand.

Gonadotrophins LH and FSH were measured by double antibody radioimmunoassay and expressed as $iu^{-1}$ of NIBSC standards 68/40 and 78/549 respectively.

Prolactin was measured by a double antibody radioimmunoassay and expressed as $mu\,l^{-1}$ of NIBSC 75/504.

All assays were subject to quality control on the Supra-Regional Assay Service Quality Control Scheme.

### *Statistical methods*

For subjects with gynaecomastia, the change in $\log_e$ E2/T ($pmol\,nmol^{-1}$) was calculated between the time just before gynaecomastia developed and the time immediately preceding this. For control patients, the time point in the course of treatment was located which corresponded most closely to that at which the gynaecomastia subjects developed the condition. This was done without knowledge of the E2/T ratios, and the change in $\log_e$ E2/T between this and the preceding sample time was calculated (Table III). Differences in the changes in the two groups of subjects were compared using a one-tailed randomisation test, since it was predicted that the group developing gynaecomastia would tend to show an increase in the circulating E2/T ratio before the condition developed.

### Results

### *Normal men*

Levels of circulating T fell with advancing age ($r = -0.68$; $P < 0.0001$), whereas there was a progressive increase in levels of $E_1$ with age ($r = 0.56$; $P < 0.0001$). Levels of $E_2$ also rose with age, but were less well correlated ($r = 0.28$; $P = 0.03$). For the normal men aged 50–80 years, the mean sex steriod levels and ranges were as follows:

Testosterone (T):

mean = 14.8 $nmol\,l^{-1}$ (2.2–41.0 $nmol\,l^{-1}$) ($n = 22$)

Oestrone (E1):

mean = 219 $pmol\,l^{-1}$ (95–417 $pmol\,l^{-1}$) ($n = 25$)

Oestradiol (E2):

mean = 182 $pmol\,l^{-1}$ (65–388 $pmol\,l^{-1}$) ($n = 26$)

### *Degree and duration of gynaecomastia*

Patients developed a mild degree of gynaecomastia lasting from 2–6 months with minimal local symptoms. In 3 cases it was unilateral (L side) and in one case bilateral. It was noticed by the subject in each case, between 4 and 13 months after beginning chemotherapy.

There was no obvious correlation between the degree and duration of the gynaecomastia and the magnitude of the preceding or associated hormone changes except in subject 6 whose gynaecomastia lasted for 6 months and in whom the greatest rises in $E_2$ were dected beforehand (Figure 1h and j ◆). This suggests that direct stimulation of breast tissue by circulating $E_2$ might have occurred. However, no hormone measurement emerged which could be used to predict subjects in whom gynaecomastia might develop during chemotherapy.

*Controls and subjects developing gynaecomastia*

Profiles of individual hormone levels, before, during and after chemotherapy are shown in Figure 1 for controls (a–e) and subjects who developed gynaecomastia (f–j). The maximum change in mean circulating sex steroid levels during chemotherapy, expressed as a percentage of basal levels, for controls and subjects developing gynaecomastia is shown in Table II.

**Table II** Maximum changes in mean circulating sex steroid level during chemotherapy, as a percentage of basal levels, for controls and subjects developing gynaecomastia.

| | *T* | *E1* | *E2* |
|---|---|---|---|
| Controls | −45% | +38% | +49% |
| Gynaecomastia | −51% | +49% | +70% |

Basal levels of testosterone in each subject were within the normal range for adult men. During chemotherapy T levels fell in every subject irrespective of whether gynaecomastia subsequently developed or not. In some subjects there was a fall to 50% or less of basal values (Figure 1a ○, ● and △). In subjects receiving chemotherapy continuously for <12 months, T levels fell progressively whilst chemotherapy was being given and rose rapidly after it was withdrawn to levels very close to basal (Figure 1a ○ and ■).

In subjects developing gynaecomastia, as a group, T levels fell further than in control subjects. This was particularly marked in two patients (Figure 1f ▽ and ▼). T levels rose rapidly after withdrawal of chemotherapy to a level exceeding basal in one subject (Figure 1f ▼), and during chemotherapy in one other subject (Figure 1f ▽). This did not occur in any other subject or control. Two subjects developed gynaecomastia during periods when circulating T levels were very low. In both subjects this resolved as T levels rose rapidly following withdrawl of chemotherapy (Figure 1f ▽ and ▼).

**Table III** $\text{Log}_e$ E2/T ratios for gynaecomastia

| *Subjects* | *Time points* | | $log_e$ *(oestradiol/testosterone)* | | |
|---|---|---|---|---|---|
| *Control* | *t3* | *t4* | *t3* | *t4* | *Difference* |
| 1. ○ | 2 | 6 | 1.812 | 2.255 | 0.443 |
| 2. ■ | 1 | 5 | 2.154 | 2.281 | 0.127 |
| 3. □ | 1 | 7 | 2.520 | 2.452 | −0.068 |
| 4. ● | 1 | 3 | 1.794 | 1.619 | −0.175 |
| 5. △ | 4 | 12 | 1.639 | 1.605 | −0.034 |
| *Gynae-comastia* | *t1* | *t2* | *t1* | *t2* | |
| 6. ◆ | 2 | 9 | 2.261 | 3.160 | 0.899 |
| 7. ▽ | 3 | 4 | 1.880 | 3.786 | 1.906 |
| 8. ▼ | 9 | 13 | 2.268 | 2.934 | 0.666 |
| 9. ▲ | 6 | 7 | 1.949 | 2.078 | 0.129 |

Shows the time (in months) after the start of treatment at which the gynaecomastia developed and the difference in the $\log_e$ (oestradiol/testosterone) ratio.

*t2* = time point immediately before gynaecomastia developed; *t1* = time point previous to *t2*; *t4* = time point corresponding to *t2* for control patients and *t3* = time point previous to *t4*.

Differences between the change in the ratio $\log_e$ E2/T just before gynaecomastia developed, compared to a corresponding change in control patients were statistically significant ($P = 0.016$).

Basal levels of $E_1$ were similar in the two groups of men. There was no significant correlation between changes in $E_1$ levels and the development of gynaecomastia.

Basal levels of $E_2$ were similar in the two groups and within the normal range for adult men. Levels in controls rose during chemotherapy, but more gradually than was the case for $E_1$. In contrast, subjects who developed gynaecomastia showed greater increases in $E_2$ during chemotherapy. Gynaecomastia developed in one subject during such an increase in $E_2$ (Figure 1h ▽), and just afterwards in two others (Figure 1h ◆ and ▲).

Statistically significant differences were found between the change in $\log_e$ E2/T just before the development of gynaecomastia, compared to corresponding times after the start of treatment in control subjects ($P = 0.016$).

Basal levels of LH and FSH were similar in the two groups of men. The administration of chemotherapy was associated with a rise of FSH levels in every subject irrespective of age, which fell again after chemotherapy was stopped.

**Figure 1** Hormone profiles for controls (a–e) and subjects developing gynaecomastia (f–j). Presence of gynaecomastia and its duration is indicated by symbols appropriate to individual subjects. (See also Table 1).

Levels of LH did not change significantly in control subjects following chemotherapy except in one man (Figure 1i ■), where LH rose during two courses of chemotherapy and fell following its withdrawal. In contrast, LH levels rose rapidly in most subjects who developed gynaecomastia and fell to basal values after withdrawal of chemotherapy (Figure 1i).

Levels of prolactin did not change significantly in any control subject during the period of the study.

SHBG levels were within the normal range and did not change significantly during or after chemotherapy in any control or subject.

Subjects developed gynaecomastia irrespective of their age at presentation with myeloma or their age at onset of chemotherapy, and older men did not develop the condition more readily than younger.

### Discussion

This is the first prospective study in males who developed gynaecomastia during treatment for cancer. The aim of this investigation was to advance our understanding of why gynaecomastia develops in some but not all men treated for multiple myeloma. The fall in T and the rise in $E_1$ and $E_2$ with age in normal men and the basal levels of the sex steroids for the nine subjects are in agreement with published data (Rubens *et al.*, 1974; Burger *et al.*, 1974). Basal levels of LH, FSH, and SHBG are also similar to published data for older men (Schalch *et al.*, 1968; Rubens *et al.*, 1974; Anderson, 1974).

We have confirmed the biochemical changes of compensated Leydig cell dysfunction induced by chemotherapy (Whitehead *et al.*, 1982). Levels of circulating T fell and $E_1$, and $E_2$, FSH and LH rose in all cases, but changes were most marked in men developing gynaecomastia, suggesting that the stimulus to breast growth was related to the severity of testicular damage. Furthermore, a significant difference between changing $E_2$/T ratios was identified between subjects with gynaecomastia and controls, which has not been shown in drug-induced gynaecomastia in previous studies. Gynaecomastia resolved when these levels returned towards normal following withdrawal of chemotherapy. It is possible, therefore, that the stimulus to breast growth is related either to an increased exposure to oestrogen, or a release from the action of T, which probably normally suppresses breast growth in men.

The origin of the increased oestrogens in our subjects remains unclear. It is known, however, that the human testis secretes $E_2$ and a small amount of $E_1$ (Baird *et al.*, 1973; Doerr & Pirke, 1974; Martikainen *et al.*, 1980). Sertoli cells also actively aromatize androgen to oestrogens following stimulation by FSH (Dorrington & Armstrong, 1975; Dorrington *et al.*, 1978; Payne *et al.* 1976). Whether this is released into the systemic circulation or is involved in intra-testicular control mechanisms is unclear.

In view of the present findings, it is possible that the initial Leydig cell damage and tubular dysfunction with consequent fall in T and rise in LH/FSH, resulted in an increase in testicular oestrogen secretion from surviving Leydig or Sertoli cells, or both. Furthermore, $E_2$ may itself regulate inhibitory enzymes on the testicular androgen biosynthetic pathway, suppressing T production still further (Saez *et al.*, 1978; Hsueh *et al.*, 1978; Huhtaniemi *et al.*, 1980). This would produce an even greater imbalance in the circulating oestrogen: androgen ratio. Approximately 50% of the blood production rate of $E_2$ arises from peripheral conversion of T (Longcope *et al.*, 1969). Whether chemotherapy affects this or the rate of excretion of androgens or oestrogens is unknown, but the possibility cannot be excluded.

Levels of SHBG did not change significantly in any subject. This was despite the considerable reduction in circulating T and the increase in $E_1$ and $E_2$ seen in some subjects who developed gynaecomastia. Clearly the magnitude and duration of these changes were insufficient to increase hepatic SHBG synthesis which might have been expected. Consequently it is unlikely that the development of gynaecomastia was related to any change in the ratio of bound:free T or oestrogens.

Both cyclophosphamide and melphalan are alkylating agents and this group of cytotoxic drugs is the most frequently recognised cause of cytotoxic-induced testicular damage (reviewed by Shalet, 1980). Our patients received both these drugs plus a certain amount of scatter irradiation to the testes, which may well have contributed to the subsequent testicular damage (Rowley *et al.*, 1974). Our study shows that following such treatment, gynaecomastia may be associated with resulting testicular damage. This further illustrates the sensitivity of male breast tissue to changes in circulating concentrations of oestrogens and testosterone.

This work was supported by a grant from the Medical Research Council. We are grateful to Dr. E. Gowland for the gonadotrophin and prolactin estimations and to Miss M. V. Kui Lee for technical help, and to the Department of Medical Illustration, University Hospital of South Manchester for the preparation of the figures.

### References

ANDERSON, D.C. (1974). Sex hormone binding globulin. *Clin. Endocrinol.* **3,** 69.

ANDERSON, D.C., HOPPER, B.R., LASLEY, B.L. & YEN, S.S.C. (1976*a*). A simple method for the assay steriods in small volumes of plasma. *Steroids,* **28,** 179.

ANDERSON, D.C., LASLEY, B.L., FISHER, R.A., SHEPHERD, J.H., NEWMAN, L. & HENDRICKZ, A.G. (1976*b*). Transplacental gradients of sex-hormone-binding-globulin in human and simian pregnancy. *Clin. Endocrinol.,* **5,** 657.

BAIRD, D.T., GALBRAITH, A., FRASER, I.S. & NEWSAM, J.E. (1973). The concentration of oestrone and oestradiol 17$\beta$ in spermatic venous blood in man. *J. Endocrinol.,* **57,** 285.

BURGER, H.G., BAKER, H.W.G., de KRETZER, D.M., HUDSON, B., FRANCHIMONT, P. & PEPPERELL, R.J. (1974). Regulation of gonadal function in the male by gonadotrcpins. in *Gonadotropins and Gonadal Failure.* (Ed. Mongdal), New York: Academic Press. p. 531.

DOERR, P. & PIRKE, K.M. (1974). Regulation of plasma oestrogen in normal adult males. *Acta Endocrinol.,* **75,** 617.

DORRINGTON, J.H., & ARMSTRONG, D.T. (1975). Follicle stimulating hormone stimulates estradiol 17$\beta$ synthesis in cultured Sertoli cells. *Proc. Natl Acad. Sc.,* **72,** 2677.

DORRINGTON, J.H., FRITZ, I.B. & ARMSTRONG, D.T. (1978). Control of testicular estrogen synthesis. *Biol. Repro.,* **18,** 55.

GALTON, D.A.G., TILL, M. & WILTSHIRE, W. (1957). Busulfan. Summary of clinical results. *Ann. N.Y. Acad. Sci.,* **68,** 967.

HSUEH, A.J.W., DUFAU, M.L. & CATT, K.J. (1978). Direct inhibitory effect of estrogen on Leydig cell function of hypophysectomized rats. *Endocrinol.,* **103,** 1096.

HUHTANIEMI, I., LEINONEN, P., HAMMOND, G.L. & VIHKO, R. (1980). Effect of oestrogen treatment on LH/hCG receptors and endogenous steroids in prostatic cancer patients. *Clin. Endocrinol.,* **13,** 561.

LARGE, D.M. & ANDERSON, D.C. (1979). Twenty-four hour profiles of circulating androgens and oestrogens in male puberty with and without gynaecomastia. *Clin. Endocrinol.,* **11,** 505.

LONGCOPE, C., KATO, T. & HORTON, R. (1969). Conversion of blood androgens to oestrogens in normal adult men and women. *J. Clin. Invest.,* **48,** 2191.

MARTIKAINEN, H., HUHTANIEMI, I. & VIHKO, R. (1980). Response of peripheral serum sex steroid and some of their precursors to a single injection of hCG in adult men. *Clin. Endocrinol.,* **13,** 157.

PAYNE, A.H., KELCH, R.P., MUSICH, S.S. & HALPERN, M.E. (1976). Intratesticular site of aromatization in the human. *J. Clin. Endocrinol. Metab.,* **42,** 1082.

ROSNER, W. (1972). A simplified method for the quantitative determination of testosterone-estradiol-binding globulin activity in human plasma. *J. Clin. Endocrinol. Metab.,* **34,** 983.

ROWLEY, M.J., LEACH, D.R., WARNER, G.A. & HELLER, C.G. (1974). Effect of graded doses of ionizing radiation on the human testis. *Radiat. Res.,* **59,** 665.

RUBENS, R., DHONT, M. & VERMEULEN, A. (1974). Further studies of Leydig cell function in old age. *J. Clin. Endocrinol. Metab.,* **39,** 40.

SAEZ, J.M., HAOUR, F., LORAS, B., SANCHEZ, P. & CATHIARD, A.M. (1978). Oestrogen induces Leydig cell refractoriness to gonadotrophin stimulation. *Acta Endocrinol.,* **89,** 379.

SCHALCH, D.S., PARLOW, A.F., BOON, R.C., REICHLIN, S. & LEE, L.A. (1968). Measurement of luteinizing hormone by radioimmunoassay. *J. Clin. Invest.,* **47,** 665.

SCHORER, A.E., OKEN, M.M., & JOHNSON, G.J. (1978). Gynaecomastia with nitrosourea therapy. *Cancer Treat. Rep.,* **62,** 574.

SHALET, S.M. (1980). Effects of chemotherapy on gonadal function of patients. *Cancer Treat. Rev.,* **7,** 141.

SMITH, R.H. & BARRETT, O. (1967). Gynaecomastia associated with vincristine therapy. *Calif. Med.,* **107,** 347.

WHITEHEAD, E.M., SHALET, S.M., BLACKLEDGE, G.A., TODD, I., CROWTHER, D., & BEARDWELL, C.G. (1982). The effects of Hodgkin's disease and combination chemotherapy on gonadal function in the adult male. *Cancer,* **49,** 418.